# Genome Sequence of *Rhodococcus* sp. Strain PML026, a Trehalolipid Biosurfactant Producer and Biodegrader of Oil and Alkanes

**DOI:** 10.1128/genomeA.00433-15

**Published:** 2015-05-07

**Authors:** C. M. Sambles, D. A. White

**Affiliations:** a School of Biosciences, Geoffrey Pope Building, University of Exeter, Exeter, United Kingdom; b Plymouth Marine Laboratory, Prospect Place, Plymouth, Devon, United Kingdom

## Abstract

*Rhodococcus* sp. strain PML026 produces an array of trehalolipid biosurfactant compounds in order to utilize hydrophobic carbon sources, such as oils and alkanes. Here, we report the high-quality draft genome sequence of this strain, which has a total length of 5,168,404 bp containing 4,835 protein-coding sequences, 12 rRNAs, and 45 tRNAs.

## GENOME ANNOUNCEMENT

Members of the *Rhodococcus* genus are well known for their ability to produce cell-bound and extracellular trehalolipid biosurfactant compounds in the presence of hydrophobic substrates (1–3). Trehalolipids exhibit a range of potential bioactivities (4–9) and are excellent emulsifying compounds with applications in microbe-enhanced oil recovery and oil spill treatment (10, 11). *Rhodococcus* sp. strain PML026 is a novel marine bacterium that was recently isolated and shown to produce a range of trehalolipid compounds in order to assimilate oil and alkanes (12). To better understand the trehalolipid production and other abilities of this strain, a genome sequence analysis of *Rhodococcus* sp. PML026 was carried out.

Using the Illumina HiSeq 2500 platform, two paired-end libraries (insert sizes, 250 bp and 500 bp) and two mate-pair libraries (insert sizes, 9 kbp and 11 kbp) were sequenced, generating 146,696,910 pairs of sequence reads (100 bp) from the two paired-end libraries and 73,808,154 read pairs (100 bp) from the two mate-pair libraries. After duplicate removal using FastUniq (13) and filtering and trimming using Trim Galore, 93% of the read pairs (136,458,888) from the paired-end libraries were retained, and 78% of the read pairs (57,494,134) from the mate-pair libraries remained. Using subsets of the two paired-end libraries, ~5 million trimmed and filtered reads per library were used for genome assembly with SPAdes (version 3.1.1) (14) using the parameter --careful with *k*-mers of 21, 33, 55, 77, 99, and 127. Further scaffolding was performed with SSPACE (version 3) (15) using all trimmed and filtered paired-end reads and mate-pair reads. Finally, GapFiller (version 1-10) (16) was used to close 19 gaps, and incorrect scaffolds were split using REAPR (version 1.0.17). The resulting assembly consisted of 37 contigs in 16 scaffolds with a total length of 5,168,404 bp. Annotation was performed using Prokka (version 1.10) (17) using a genus database generated from four *Rhodococcus* sp. genomes, *R. erythropolis* PR4 (GenBank accession no. NC_012490), *R. jostii* RHA1 (GenBank accession no. NC_008268), *R. opacus* B4 (GenBank accession no. NC_012522), and *R. pyridinivorans* SB3094 (GenBank accession no. NC_023150). tRNAs and transfer-messenger RNAs (tmRNAs) were predicted using Aragorn (version 1.2), ribosomal RNAs with Barrnap (version 0.5), and coding sequences with Prodigal (version 2.60). The genome contains 4,835 protein-coding sequences, 12 rRNAs, and 45 tRNAs.

### Nucleotide sequence accession number.

The genome sequence and annotation data for *Rhodococcus* sp. PML026 have been submitted to GenBank under the accession no. JZIS00000000.
